# Assessment of endogenous insulin secretion in insulin treated diabetes predicts postprandial glucose and treatment response to prandial insulin

**DOI:** 10.1186/1472-6823-12-6

**Published:** 2012-06-08

**Authors:** Angus G Jones, Rachel EJ Besser, Beverley M Shields, Timothy J McDonald, Suzy V Hope, Bridget A Knight, Andrew T Hattersley

**Affiliations:** 1 Peninsula NIHR Clinical Research Facility, Peninsula Medical School, University of Exeter, Exeter, UK; 2 Royal Devon and Exeter Hospital, Barrack Road, Exeter, EX2 5DW, UK

**Keywords:** Diabetes, C-peptide, Postprandial, Glucose, Insulin

## Abstract

**Background:**

In patients with both Type 1 and Type 2 diabetes endogenous insulin secretion falls with time which changes treatment requirements, however direct measurement of endogenous insulin secretion is rarely performed. We aimed to assess the impact of endogenous insulin secretion on postprandial glucose increase and the effectiveness of prandial exogenous insulin.

**Methods:**

We assessed endogenous insulin secretion in 102 participants with insulin treated diabetes (58 Type 1) following a standardised mixed meal without exogenous insulin. We tested the relationship between endogenous insulin secretion and post meal hyperglycaemia. In 80 participants treated with fast acting breakfast insulin we repeated the mixed meal with participants’ usual insulin given and assessed the impact of endogenous insulin secretion on response to exogenous prandial insulin.

**Results:**

Post meal glucose increment (90 minute - fasting) was inversely correlated with endogenous insulin secretion (90 minute C-peptide) (Spearman’s r = −0.70, p < 0.001). Similar doses of exogenous prandial insulin lowered glucose increment more when patients had less endogenous insulin; by 6.4(4.2-11.1) verses 1.2(0.03-2.88) mmol/L (p < 0.001) for patients in the lowest verses highest tertiles of endogenous insulin.

**Conclusions:**

In insulin treated patients the measurement of endogenous insulin secretion may help predict the degree of postprandial hyperglycaemia and the likely response to prandial insulin.

## Background

Guidelines for treatment in Type 1 and Type 2 diabetes differ greatly predominantly reflecting differences in endogenous insulin secretion [1-3]. Within both major subgroups of diabetes there is both between individual variation and with time intra-individual reduction in a patient’s endogenous insulin secretion which results in differing treatment requirements [4-6]. Traditionally, in clinical practice, endogenous insulin secretion is not measured and treatment decisions are made on the basis of glycaemic control and clinical diagnosis of diabetes subtype. There is some evidence supporting direct measurement of endogenous insulin secretion to assess the most appropriate treatment for a patient, particularly in the context of predicting response to oral therapy [7-19]. Little is known regarding whether measuring endogenous insulin secretion can assist choice of insulin regimen.

It is possible to measure endogenous insulin secretion in clinical practice using C-peptide, which is secreted in equimolar amounts to insulin [20]. 90 minute C-peptide in a formal mixed meal test is a robust assessment of insulin response in insulin treated patients [21].

One area where underlying insulin secretion is likely to affect treatment requirements is requirement for prandial exogenous insulin. Intensive insulin regimens with prandial rapid or short acting insulin are clearly appropriate in Type 1 diabetes outside the honeymoon period where there is absolute insulin deficiency [3]. However in Type 2 diabetes where endogenous insulin secretion is preserved, excellent glycaemic control can be achieved using basal (intermediate or long acting) insulin without rapid or short acting prandial insulin [22,23].

We hypothesised that in insulin treated diabetes, patients with higher endogenous insulin secretion will have a lower rise in glucose after meals and will respond less to prandial insulin. We aimed to assess this in a mixed population of Type 1 and Type 2 diabetes with a wide spectrum of insulin secretion.

### Aims

To assess the relationship between endogenous insulin secretion as measured by 90 minute post mixed meal serum C-peptide and:

1. Post-prandial glucose increment (90 minute – fasting glucose) in a standardised mixed meal test without concurrent exogenous prandial insulin

2. Treatment response to exogenous prandial insulin as assessed by change in mixed meal glucose increment when exogenous prandial insulin is given

## Methods

### Study participants

We recruited 102 adults with insulin treated diabetes, HbA1c <86 mmol/mol (10%) and without renal impairment (eGFR > 60mls/min/1.73 m^2^) from existing research databases and clinical secondary care, as described previously [24,25]. 58 had Type 1 diabetes (16 within 3 years of diagnosis, median (interquartile range, IQR) age of diagnosis 20 (14–27), BMI 25 (22–27)), 44 had Type 2 diabetes (median (IQR) age of diagnosis 55 (47–59), BMI 29(28–36), classification based on clinical diagnosis from health records), 60 were male. Median (IQR) age was 57 years (42–69), diabetes duration 16 years (6–28), BMI kg/m^2^ 27 (24–29) and HbA1c 63 mmol/mol (55–72) (7.9% (7.2-8.7)). The study was approved by the South West Research Ethics Committee (UK) and conducted in accordance with the Declaration of Helsinki. Written informed consent was obtained from all participants.

### Mixed meal tests

All patients underwent a standardised morning mixed meal test (MMT) without morning insulin. In a subgroup of 80 patients treated with prandial breakfast insulin (rapid analogue 61 (4 via insulin pump), rapid analogue/basal mixed 9, human prandial soluble 2, human soluble/basal mixed 8) a further morning MMT was performed with participants’ normal morning insulin dose given. Mixed meal tests were conducted in random order using a randomization list generated in StatsDirect 4 (StatsDirect Ltd, UK), between 48 h and 2 weeks apart. In addition all participants collected a home urine sample 2 hours after their largest meal for urine C-peptide creatinine ratio (UCPCR) as described previously [24-26].

### Mixed meal test without insulin (MMT)

This was performed according to a standard protocol as reported previously [21,25]. In brief participants fasted from midnight without taking their usual morning insulin or OHA. Capillary glucose was measured pre test and test rescheduled if <4 or >15 mmol/L (<72 or >270 mg/dl). Serum C-peptide, creatinine, glucose, and HbA1c were measured on a fasting sample. Participants consumed a standardised mixed meal (Ensure Plus HP (Abbott Nutrition, Illinois, USA) 6 ml/kg (maximum 360 ml), content per 100 ml: carbohydrate 15.9 g, protein 7.9 g, fat 3.3 g, energy 125 kcal). C-peptide and glucose were measured at 90 minutes post completion of mixed meal.

### Mixed meal test with insulin (MMT + I)

Performed as per mixed meal protocol above except participants took their usual morning insulin dose before ingestion of the mixed meal. Participants were asked not to correct for hyperglycaemia. Investigators advised a reduced insulin dose in 9 participants where home breakfast was judged to contain substantially more carbohydrate than the mixed meal. Those carbohydrate counting (including insulin pump users) used their normal breakfast insulin to carbohydrate ratio. Normal basal insulin was continued in all participants. Oral hypoglycaemic medications were withheld until completion of the MMT on the morning of both tests.

### Sample analysis

All samples were analysed in the Biochemistry department at the Royal Devon & Exeter Hospital, Exeter, UK. We undertook C-peptide analysis using the routine automated Roche diagnostics (Manheim, Germany) E170 immuno-analyser.

### Statistical analysis

Data were not normally distributed therefore non parametric tests were used. We assessed the relationship between 90 minute serum C-peptide (SCP) in MMT and both glucose increment (90 minute glucose minus fasting glucose) in MMT and decrease in glucose increment with concurrent insulin (increment in MMT minus increment in MMT + I). We used Spearman’s rank correlation coefficient to assess correlations. Linear regression analysis was used to assess the magnitude of these relationships and to adjust for potential confounders. Residuals were checked to ensure model assumptions were met, given the data were not normally distributed.

In the participants who completed MMT + I data were split into tertiles of endogenous insulin secretion defined by 90 minute serum C-peptide. We assessed statistical trends in mixed meal test results and participant characteristics across tertiles of endogenous insulin secretion using the Jonckheere test or (for proportions) Chi-squared for trend.

## Results

### Patients who have less endogenous insulin secretion have a higher glucose increase after a mixed meal

**Figure 1:**
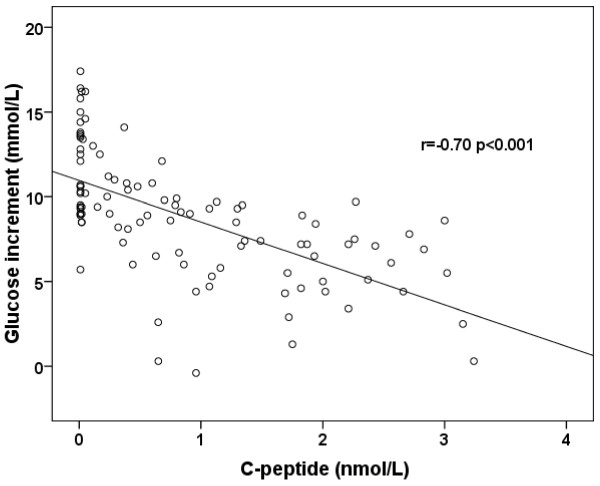
**Relationship between MMT stimulated C-peptide (nmol/L) and glucose increment (90 minute glucose – fasting glucose, mmol/L) in MMT without concurrent insulin.** r = Spearmans ro correlation coefficient.

The glucose increment in a mixed meal, defined as the increase in glucose from fasting to 90 minutes post meal (90 minute – fasting glucose) was negatively correlated with 90 minute serum C-peptide (SCP) (Spearman’s r = −0.70, p < 0.001, Figure 1) indicating that the glucose increment was smaller with higher C-peptide. In line with this, linear regression showed the association was consistent with a fall of 2.4 mmol/L glucose for every 1 nmol/L increase in SCP (B = −2.4 (CI −3.1 to −1.8, p < 0.001)). This was also shown by analysing glucose response by tertiles of endogenous insulin secretion where glucose increment was greatest in lower tertiles of endogenous insulin secretion; median (IQR) glucose increment 11.4 mmol/L (9.4-14.0) tertile 1, 9.0 (6.4-10.5) tertile 2 and 7.0 (4.6-8.0) tertile 3, p < 0.001.

### Patients with less endogenous insulin secretion have greater response to exogenous prandial insulin

**Figure 2:**
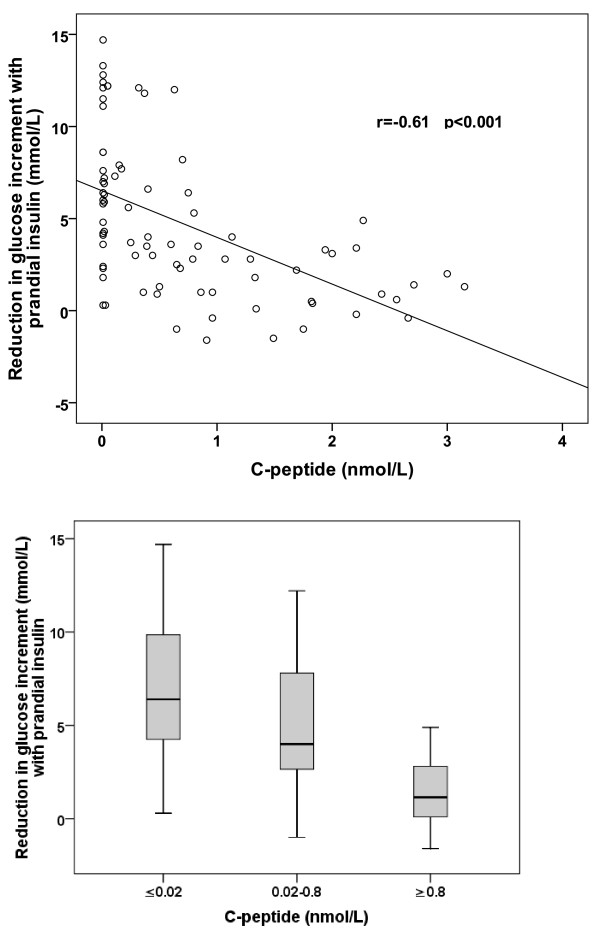
**a: Scatterplot showing the relationship between MMT stimulated C-peptide (nmol/L) and reduction in glucose increment with administration of prandial exogenous insulin.b: Boxplot showing reduction in MMT glucose increment with the addition of prandial exogenous insulin by 90 minute post MMT C-peptide tertile.** Horizontal line represents median, box interquartile range, ‘whiskers’ represent spread of remaining values. p for trend <0.001.

To assess the impact of prandial exogenous insulin we measured the reduction in glucose increment after a mixed meal when prandial exogenous insulin was given (glucose increment in MMT minus glucose increment in MMT + I). The reduction in glucose increment with administration of prandial exogenous insulin was also negatively correlated with SCP (r = −0.61, p < 0.001, n = 80, Figure 2A). Exogenous prandial insulin resulted in a greater reduction in glucose increment in those with lower C-peptide; linear regression B was −2.5 (CI −3.4 to −1.6, p < 0.001) suggesting a fall of 2.5 mmol/L in the reduction in glucose increment with prandial insulin administration for every 1 nmol/L increase in SCP. The relationship persisted after adjusting for age, age of diagnosis, BMI, gender, fasting glucose and HbA1c (p = 0.04). Of these clinical variables in multivariable analysis only SCP and age of diagnosis (p = 0.02) were statistically significant predictors of response to exogenous prandial insulin.

To further assess this relationship we subdivided our participants by tertiles of endogenous insulin secretion. Characteristics of participants in each tertile are shown in Table 1 and mixed meal results by tertile in Table 2. Patients in the lowest tertile of endogenous insulin secretion were likely to be diagnosed younger and have lower BMI, consistent with a greater proportion having type 1 diabetes, but had similar HbA1c compared with the other tertiles. The associated reduction in glucose increment with prandial exogenous insulin was substantially lower with increasing C-peptide; median (IQR) 6.4 mmol/L (4.2-11.1) tertile 1, 4.0 (2.5-7.9) tertile 2 and 1.2 (0.03-2.88) tertile 3 (p < 0.001), Figure 2B. This occurred despite similar insulin doses and glycaemic control (Table 1).

**Table 1:** Characteristics of participants (median (IQ range)) by 90 minute MMT stimulated C-peptide (SCP, nmol/L) tertile

	**Tertile 1 (SCP ≤ 0.02,n = 27)**	**Tertile 2 (SCP 0.02-0.8, n = 27)**	**Tertile 3 (SCP ≥ 0.8, n = 26)**	**p for trend**
Age (years)	53 (42–62)	42 (30–64)	62 (37–71)	0.70
Age of diagnosis (years)	15 (8–23)	27 (21–44)	50 (28–59)	**0.001**
Duration of Diabetes	36 (26–45)	16 (3–21)	10 (2–15)	**0.001**
BMI	26 (23–28)	25 (23–29)	29 (25–37)	**0.013**
HbA1c (%)	7.9 (7.2-8.7)	7.7 (7.0-8.7)	7.9 (7.4-8.7)	0.57
HbA1c (mmol/mol)	63 (55–72)	60 (53–71)	63 (57–72)	0.57
Proportion Type 1 diabetes	93%	78%	31%	**0.001**
Fasting glucose (mmol/L) (Test 1)	10.1(7.9-12.9)	8.8 (6.4-12.8)	8.4 (7.0-10.5)	0.064
90 Minute post MMT C-peptide (nmol/L)	0.01 (0–0.01)	0.40 (0.23-0.65)	1.79 (1.12-2.30)	**0.001**
Prandial (rapid or soluble only*) insulin dose administered in MMT+I (units)	8 (5–10)	8 (6–12)	6.3 (4.8-12.3)	0.98
Total daily insulin dose (units)	46 (33–64)	42 (28–62)	45 (26–88)	0.90

Participants completing both MMT and MMTI only.

* where participant took a mixed morning insulin the rapid/soluble component was calculated.

**Table 2:** Mixed meal test results (median (IQ range)) by 90 minute MMT stimulated C-peptide (SCP, nmol/L) tertile

	**Tertile 1 (SCP ≤ 0.02,n = 27)**	**Tertile 2 (SCP 0.02-0.8, n = 27)**	**Tertile 3 (SCP ≥ 0.8, n = 26)**	**p for trend**
Glucose increment in MMT (without insulin, mmol/L)	10.6 (9.0-13.7)	9.9 (8.2-11.0)	7.1 (4.4-8.9)	**0.001**
Glucose increment in MMT+I (insulin given, mmol/L)	4.3 (0.4-8.8)	4.4 (2.2-4.4)	5.2 (3.6-6.4)	0.45
Reduction in glucose increment when prandial insulin given (mmol/L): all participants (n = 80)	6.4 (4.2-11.1)	4.0 (2.5-7.9)	1.2 (0.03-2.88)	**0.001**
Reduction in glucose increment when prandial insulin given (mmol/L): Rapid analogue insulin only* (n = 61):	6.7 (4.3-11.2)	4.0 (2.3-7.9)	1.4 (1.8-3.0)	**0.001**
Reduction in glucose increment when prandial insulin given (mmol/L): Type 1 only (n = 54)	6.9 (4.2-11.3)	4.0 (2.4-7.8)	1.9 (0.3-3.5)	**0.002**
Reduction in glucose increment when prandial insulin given (mmol/L): Type 2 only (n = 26):	5.1 (4.2-6)	4.5 (2.4-9.2),	1.1 (−0.3-2.4)	**0.006**

Participants completing both MMT and MMTI only.

*excluding premixed insulin.

### These associations remain in those with the same type of diabetes and similar insulin treatment

Our results were not simply due to differences in diabetes subtype or prandial exogenous insulin type. In those treated with rapid analogue prandial insulin (basal bolus/pump regimen only - analogue mixed insulin excluded, n = 61) the correlation between 90 minute C-peptide and reduction in glucose increase with prandial exogenous insulin (r = −0.56, p < 0.001) and the relationship across tertiles of endogenous insulin secretion (Table 2) were maintained. In those with the same type of diabetes the correlations (type 1 diabetes r = −0.49 (p < 0.001, n = 54), type 2 diabetes r = −0.41 (p = 0.037, n = 26)) and the relationship across tertiles (Table 2) were also maintained. Results did not differ when analysed by gender.

### Fasting serum C-peptide and home post-prandial UCPCR can be used as the assessment of endogenous insulin secretion

When insulin secretion was assessed by other measurements similar relationships were seen.

When assessed by the measurement of fasting C-peptide correlation with postprandial glucose increase was −0.69 (p < 0.001) and correlation with reduction in glucose increment with exogenous prandial insulin −0.59 (p < 0.001). The values of fasting C-peptide defining tertiles of endogenous insulin secretion were <0.02, 0.02-0.28 and >0.28 nmol/L. The associated fall in glucose increment with prandial insulin was [median (IQR)] 6.9 mmol/L (4.2-11.5) tertile 1, 3.6 (2.3-7.3) tertile 2 and 1.4 (0.4-3.3) tertile 3 (p < 0.001).

When assessed by the measurement of UCPCR correlation with postprandial glucose increase was −0.59 (p < 0.001) and correlation with reduction in glucose increment with exogenous prandial insulin −0.51 (p < 0.001). The values of home evening meal UCPCR defining tertiles of endogenous insulin secretion were <0.03, 0.03-0.73 and >0.73 nmol/mmol. The associated fall in glucose increment with prandial insulin was [median (IQR)] 6.4 mmol/L (4.1-9.4) tertile 1, 4.0 (1.3-7.9) tertile 2 and 2.0 (0.5-3.3) tertile 3 (p < 0.001).

## Discussion

We have shown that patients with less endogenous insulin secretion have greater post meal hyperglycaemia and greater response to prandial exogenous insulin.

In our study of insulin treated patients with Type 1 and Type 2 diabetes postprandial hyperglycaemia is inversely related to endogenous insulin secretion. This is consistent with previous research in non insulin treated Type 2 diabetes has shown that those with low insulin secretion have higher glucose increment after oral glucose tolerance test and higher glycaemic variability [27,28]. In Type 1 diabetes a reduction in glycaemic variability with restoration of even small amounts of residual insulin secretion has been demonstrated after islet transplantation [29].

To our knowledge this is the first study to examine the relationship between endogenous insulin secretion and the impact of prandial exogenous insulin administration. We showed that the glucose response to exogenous prandial insulin was greatest in those with the lowest endogenous insulin secretion. In those with the highest endogenous insulin secretion concurrent prandial exogenous insulin had little effect on glucose response despite similar insulin doses and glycaemic control. A probable explanation is that those with high endogenous insulin secretion are exposed to their own endogenous prandial insulin therefore exogenous prandial insulin may be only a small proportion of their total prandial insulin exposure. This is in contrast to those with little endogenous insulin secretion where exogenous insulin makes up their entire prandial insulin exposure. In addition the similar exogenous insulin doses and glycaemic control across tertiles of endogenous insulin secretion mean insulin resistance is likely to be higher in those with preserved insulin secretion.

Our findings have potential practical implications for the management of diabetes. Patients with Type 2 diabetes progressively lose their beta-cell function over time leading to increased treatment requirements[6]. Rates of progression vary widely and progression to severe insulin deficiency may occur rapidly where a patient with LADA or Type 1 diabetes has been misclassified as Type 2[30,31]. A large proportion of patients with Type 2 diabetes will eventually require insulin. While background insulin alone may achieve initial glycaemic control many patients progress to prandial insulin treatment. The decision when to change to prandial insulin is not well defined and endogenous insulin secretion is rarely measured[32]. Our work suggests that prandial insulin will be most effective when patients have a lower level of endogenous insulin as defined by a C-peptide below the top tertile of our participants; 90 minute post MMT C-peptide <0.8 nmol/L: fasting C peptide <0.29 nmol/L, or UCPCR 2 hours post home evening meal <0.73 nmol/mmol. Prandial fast acting exogenous insulin may have little impact on post meal hyperglycaemia above these levels. Assessment of endogenous insulin secretion could also potentially assist in decisions on rationalising a patient’s insulin regimen, for example where there are potential difficulties with administering multiple insulin doses or where adherence is thought to be poor a move to once or twice daily basal insulin may be justified where endogenous insulin secretion is preserved.

Limitations of our study include that we have recruited participants with a mixture of Type 1 and Type 2 diabetes and that differences seen may reflect differences in insulin secretion between the two diabetes subtypes. However the relationships seen, although weaker, remains when analysing our results by type of diabetes suggesting the relationship between insulin secretion and glucose response is not due to differences between diabetes subtypes alone. It is possible that results could have been influenced by differences in participant’s oral hypoglycaemic agents. While diabetes treatments were withheld on the morning of mixed meal tests, residual levels of treatment taken the previous day could potentially still affect both glucose and C-peptide. However this is unlikely to systematically differ between mixed meal tests (which were conducted in random order) and a longer period without medication might have made results less applicable to clinical practice.

A further potential limitation is that our marker of insulin secretion (90 minute post MMT C-peptide) and measure of post meal glucose increase (glucose increase from 0 to 90 minutes in the MMT) were measured within the same test. Our findings are strengthened by the demonstration of similar results when using fasting C-peptide and UCPCR (measured on a separate occasion) as markers of insulin secretion. The MMT, while well established and likely more reproducible than a conventional meal, may not be a normal physiological stimulus. The more rapid absorption of a liquid meal could lead to an earlier and greater glucose peak than a non liquid meal with the same carbohydrate content. In addition a standardised meal may not reflect a person’s normal intake. The liquid meal given is likely to have higher carbohydrate content than many participants normal breakfast (the carbohydrate content of our mixed meal was 57 g in those >60 kg, equivalent to 3–4 pieces of toast).

While we have shown a clear relationship between insulin secretion and effectiveness of fast acting (prandial) exogenous insulin during a mixed meal test further studies are needed to assess whether C-peptide could be a clinically useful measure to assist choice of insulin regimen. It may be that direct measurement of insulin secretion may assist clinical decisions on optimal insulin treatment. While a formal mixed meal test is unlikely to be an option in mainstream clinical practice the association was preserved using fasting C-peptide and post home meal UCPCR which may be more applicable to clinical practice [26].

## Conclusion

Endogenous insulin secretion is predictive of postprandial hyperglycaemia and response to prandial exogenous insulin. The measurement of endogenous insulin secretion may be a helpful guide to insulin therapy.

## Competing interests

The authors declare that they have no competing interests.

## Authors’ contributions

AGJ, REJB, BAK,TJM and ATH participated in study design, AGJ, REJB, BAK and SVH collected data, AGJ, REJB & BMS performed data analysis, all authors participated in drafting or revising the manuscript and approved the final manuscript.

## Pre-publication history

The pre-publication history for this paper can be accessed here:

http://www.biomedcentral.com/1472-6823/12/6/prepub
